# Signs of oral dryness in relation to salivary flow rate, pH, buffering capacity and dry mouth complaints

**DOI:** 10.1186/1472-6831-7-15

**Published:** 2007-11-09

**Authors:** Najat MA Farsi

**Affiliations:** 1 Department of Preventive Dental Sciences, Faculty of Dentistry, King Abdulaziz University, PO Box 80209, Jeddah 21089, Saudi Arabia

## Abstract

**Background:**

This study aimed to investigate the signs of oral dryness in relation to different salivary variables and to correlate subjective complaints of oral dryness with salivary flow rate.

**Methods:**

312 unmedicated healthy individuals belonging to three age groups, (6–11, 12–17, and 18–40 years) were examined clinically for signs of oral dryness. Resting and stimulated saliva were collected to determine flow rate, pH and buffering capacity. A questionnaire was used to obtain information on subjective sensation of dry mouth.

**Results:**

Dry lip and dry mucosa were present in 37.5% and 3.2% of the sample respectively. The proportion of subjects who complained of oral dryness (19%) showed a stimulated salivary flow rate significantly lower than non complainers. Dry lip was significantly related to low resting flow rate but pH and buffering capacity did not show any significant relation to dry lip. Dry mucosa was not related to any of the above mentioned parameters.

**Conclusion:**

The finding that the stimulated salivary flow rate was reduced in subjects complaining of dry mouth is of great clinical relevance, since the reduction is expected to be reflected in compromising various salivary functions.

## Background

In healthy individuals the oral tissues are constantly bathed with saliva. The constant flow of saliva eliminates food debris and bacteria by swallowing. When stimulated by chewing or by moderate strength citric acid, the whole saliva flow rate increases from the resting value of around 0.3–0.65 ml/min to around 1.5–6.0 ml/min [1,2] with great individual variations.

Saliva has an important role in oral health by providing immunological protection, and its role as a reservoir of ions which aid remineralization of the calcified tissues [3]. The secretions of salivary glands are important for protecting the mucosa from feeling dry and according to Naito et al this oral dryness is contributing to reduce health related quality of life [4]. The perception of oral dryness (xerostomia) was found to be related to the rate of secretion of minor palatal glands [5,6]. The feeling of dryness was evident when the rate of that secretion was below 3 μL/cm^3^/min [7]. This suggests that the sensation of a dry mouth is perceived when there is insufficient mucosal wetting, especially of the palate. Others considered that the subjective feeling of oral dryness might be a result of salivary gland hypofunction [8,9]. Hence, from the clinical point of view there will be a considerable difficulty in making a decision as to whether a given patient has salivary gland hypofunction, a condition that requires additional salivary gland evaluation. Associating saliva flow rate with other clinical features such as dryness of the lip, dryness of the oral mucosa and difficulty with speech and swallowing may aid in such decision making process [10,11].

Other than salivary hypofunction, a dry mouth can be caused by variety of reasons: dehydration, mouth breathing, medications and radiotherapy [12].

Although numerous studies discussed functional disturbance of salivary gland in relation to xerostomia in elderly people [13-15], it appears that very few studies included children and adolescents [16,17]. The prevalence of oral dryness, among different age groups despite its effect on oral health, has not been elucidated in many populations, including Saudi Arabians.

Many studies have estimated values of stimulated salivary flow and found them to be normal in subjects complaining of xerostomia [18]. However, since the resting saliva is actually the flow that occurs continuously in the patients mouth, this flow would be more relevant and may correlate better with patients symptoms, especially in cases where the gland can be stimulated to produce enough saliva. The present study was planned to investigate whether resting or stimulated flow correlate better with symptoms and signs of oral dryness.

This study aimed to determine: (i) the prevalence of signs of oral dryness among 3 groups of children, adolescents and adults; (ii) whether signs of oral dryness are related to the salivary variables namely flow rate, pH, and buffer capacity; (iii) whether salivary flow rate is correlated to subjective complaints of oral dryness.

## Methods

### Study population

Using the random number table, 312 outpatients attending screening clinics at the school of dentistry, King Abdulaziz University, were selected according to their serial number. Participants age ranged from 6 to 40 years. The sample was then stratified according to age into 3 groups. The age and sex distribution of the participants are shown in Table 1. Study subjects were requested to answer questionnaire regarding medication intake and general medical data. Only healthy unmedicated individuals were included in the study. Symptoms of oral dryness were recorded with modification according to Bardow et al [19]. Using the following questions:

**Table 1:** Distribution of dry lip and mucosa

**Age (years)**	**Gender**	**N**	**Mean age**	**Dry lip**		**Dry mucosa**	
				
				**Yes**	**%**	**Yes**	**%**
**6 – 12**	M	46	9.1	18	39.1	0	0
	F	68	8.8	20	29.4	2	3.0
	Total	114	8.9	38	33.3	2	1.8
**13 – 18**	M	44	16.3	17	38.6	3	6.8
	F	55	15.7	27	49.1	1	1.8
	Total	99	15.9	44	44.4	4	4
**19 – 40**	M	43	32.1	22	51.2	2	4.8
	F	56	31.7	13	23.2	2	3.6
	Total	99	31.3	35	35.3 *	4	4.1
**TOTAL**	M	133	19.2	57	42.8	5	3.1
	F	179	18.7	60	33.5	5	4.3
	Total	312	18.8	117	37.5	10	3.2

* P value of sex difference is 0.01

Q1. Does your mouth feel dry?

Q2. Do you sip liquids to aid in swallowing dry food?

Q3. Does your mouth feel dry when eating a meal?

Q4. Does the amount of saliva in your mouth seem to be too little?

Subject who answered affirmatively to at least one of the questions related to oral dryness were considered as positive for subjective complaints of oral dryness

The ethical committee, at the school of dentistry, approved the study, and all the patients or their guardians signed an informed consent form.

### Data collection

The subjects were scheduled for the screening clinic at the school of dentistry and underwent clinical examination, sialometry and analysis of the saliva. Data were collected by two experienced dentists. Prior to the start of the study, the examiners held series of sessions to standardized data collection technique and reached to good levels of intra-and inter-examiners reliability. The reliability levels varied from 0.81 to 0.92 using Cohen's Kappa statistics.

### Dryness of lip and oral mucosa

Signs of oral dryness were registered clinically by clinical observation of lip and oral mucosa dryness. Oral mucosa dryness was recorded when the investigator noticed the absence of a saliva coating over most of the dorsum of the tongue, buccal and labial mucosa as well as the absence of pooled saliva in the floor of the mouth [20]. Lip dryness was recorded when either upper or lower lip lacked the characteristic shiny appearance and or in presence of chapped lip.

### Salivary analysis

After oral examination, whole mouth saliva was analyzed as described by previous studies [21,22]. The following variables were recorded.

#### Flow rate

Both resting and stimulated saliva were collected from the study population between 9:00 to 12:00 noon no earlier than 2 hours after meal. Prior to collection of each sample the subject was asked to sit down and relax. Unstimulated saliva was collected first for 5 minutes. Following, paraffin stimulated saliva was collected for an additional 5 minutes. The saliva was collected in a graduated sampling tube fitted with a funnel.

#### pH value

The pH of the collected resting and stimulated saliva was measured, immediately after collection, using EC 40 Benchtop pH/ISE meter (product of Hach company). To measure the pH of the saliva 0.1 ml of saliva was dropped onto the pH-sensitive electrode. The digital reading was allowed to stabilize for a few seconds before the pH reading was taken. Autocalibration is a feature of the EC40 meter that automatically recognizes five pH buffers: 1.68, 4.01, 7.00, 10.01, and 12.46, within a range of ± 0.5 pH units.

#### Buffering capacity

Saliva buffering capacity was assessed immediately after the collection using a commercial Dentobuff strip test (CRT Buffer, vivadent). The buffer effect was determined by comparing visually the color changes in the dentobuff strip employing the manufacturer's colour chart. The buffering capacity was classified as 1 = low, 2 = intermediate or 3 = high.

### Statistical analysis

Statistical analysis was performed using SPSS program. Differences between groups were tested using t-test and Pearson chi square. When the expected value was less than 5, Fisher exact test was complemented. The level of significance was set at p < 0.05.

## Results

Upon clinical examination, the prevalence of dry lip in the total sample was estimated to be 37.5% (table 1). Dry lip was observed in 33.3, 44.4 and 35.3% of children, adolescent and adults respectively. The difference between the three age groups was not statistically significant. The sex difference was statistically significant in the adults group, where dry lip occurred in 51.2% of the males in contrast to 23.2% of the females.

Dry mucosa was less evident among the study groups than dry lip, it was prevalent in 3.2% of the sample with no age or sex differences.

Data on lip and mucosa dryness in relation to resting saliva parameters is presented in Table 2. The mean resting saliva flow rate was 0.65 ml/min in subjects with dry lip. This mean was higher (0.73 ml/min) in subjects with normal lip. The difference in resting saliva flow rate between subjects with and without dry lip was the only statistically significant difference. Other parameters failed to show any significant association with signs of dry lip or mucosa.

**Table 2:** Signs of oral dryness in relation to resting saliva parameters

**Saliva parameters**			**Total **N = 312	**Dry lip**		**Dry mucosa**	
				
				**Present **N = 117	**Absent **N = 195	**Present **N = 10	**Absent **N = 302
**Flow rate**	Mean		0.68	0.65	0.73*	0.63	0.68
	SD		0.62	0.65	0.57	0.71	0.63
**PH**	Mean		7.11	7.11	7.12	7.10	7.11
	SD		0.37	0.39	0.41	0.35	0.38
**Buffer Capacity**	1	n	95	37	55	3	85
		%	30	32	28	30	28
	2	n	180	65	108	5	163
		%	58	56	55	50	54
	3	n	37	17	32	2	54
		%	12	15	17	20	18

* Using t test, P value of difference between groups is 0.02

Table 3 presents the relationship between stimulated saliva parameters and signs of dry lip and mucosa. None of the parameters showed any significant relationship with dry lip or mucosa.

**Table 3:** Signs of oral dryness in relation to stimulated saliva parameters

**Saliva parameters**			**Total **N = 312	**Dry lip**		**Dry mucosa**	
				
				**Present **N = 117	**Absent **N = 195	**Present **N = 10	**Absent **N = 302
**Flow rate**	Mean		1.53	1.51	1.55	1.43	1.53
	SD		1.1	1.3	1.2	1.0	1.4
**pH**	Mean		7.39	7.38	7.40	7.38	7.39
	SD		0.39	0.41	0.46	0.43	0.41
**Buffer Capacity**	1	n	7	3	6	0	12
		%	2	2	3	0	4
	2	n	71	29	40	4	75
		%	23	25	21	40	25
	3	n	234	85	149	6	215
		%	75	73	76	60	71

Table 4 shows the means and standard deviations of saliva flow rate in subjects who responded positively and negatively to questions regarding their complaints. Sixty subjects (19.2%) answered yes to at least one question. The stimulated saliva flow rate was significantly related to subjective complaints of dryness. This association was not evident when considering the resting flow rate. The answer to the question regarding liquid sipping to aid swallowing dry food was the one mostly significant complaint in relation to reduced stimulated saliva flow rate.

**Table 4:** Subjective complaints of oral dryness in relation to resting and stimulated salivary flow rates.

		**Saliva flow rate**					
		
**Questions**		**Resting**		**P value**	**Stimulated**		**P value**
		**Mean**	**SD**		**Mean**	**SD**	
**Q1**	**Yes **63	0.67	0.5	NS	1.5	0.7	0.02
	**No **249	0.69	0.7	NS	1.7	0.8	NS
**Q2**	**Yes **60	0.65	0.4	NS	1.4	0.6	0.00
	**No **252	0.70	1.1	NS	1.8	0.9	NS
**Q3**	**Yes **61	0.64	0.4	NS	1.4	0.6	0.01
	**No **251	0.69	1.1	NS	1.7	0.8	NS
**Q4**	**Yes **64	0.68	0.5	NS	1.4	0.6	0.01
	**No **248	0.68	0.7	NS	1.6	0.8	NS

P value using t test

NS = non significant

## Discussion

Xerostomia was defined as the subjective sensation of dry mouth, while hypo salivation was known as the objective finding of a reduced salivary flow rate [23]. In the present study 37.5% of the samples had dry lip and 3.2% of them had dry mucosa.

Dry lip was the most prevalent sign of oral dryness in this study probably because it is the site of initial contact with inspired air. It was reported that the anterior part of the palate and anterior dorsum of the tongue are the sites where xerostomia symptoms more pronounced [7] because saliva is very susceptible to evaporation from those areas during mouth-breathing. In Saudi Arabia, especially in Jeddah, the very hot weather could be the reason behind excessive dryness of the lips. Since veil constitutes an important component of dress for women in Saudi Arabia, it is expected to see females with less lip dryness than males.

It was reported that physiological pH range increases as the flow rate increases and vice versa, thereby maintaining a saliva pH within a range of minimum 6.6 for resting saliva to 7.4 for stimulated saliva was recorded as acceptable [24]. The present study investigated signs of oral dryness in relation to salivary flow rate, pH and buffering capacity and found that subjects with lip dryness exhibited reduction in resting salivary flow rate more often than did subjects who had normal lip. However, changes of pH and buffering capacity were not of significant values. Wolff and Klienberg [6] found that saliva flow rate and pH were both directly related to mucosal wetness. In this study data on dry mucosa which was recorded only in ten subjects could not be conclusive.

The present study used self administered questionnaire that included questions which are predictive of reduced saliva output. Question number one related to the resting saliva production because it focused on the patient's general feeling of oral dryness. The rest of the questions were mainly oriented at the stimulated saliva production to assist in chewing and swallowing. Complaints of dry mouth were reported by 19% of the sample. This result is comparable to that reported earlier among another group from central region of Saudi Arabia [25]. Studies of xerostomia complaints in elders reported prevalence that varied from 28 to 63% [26-29]. Most of those studies used subjects residing in hospitals and community dwelling. One study examined 529 elders out patients and reported that 29% of the entire group had complaints of oral dryness [12].

In the present study, complaints of dry mouth are lower than previous studies which did not indicate the proportion of subjects who had an underlying disease. Although the disagreement might reflect racial, social and culture differences between the samples, it may also be attributed to the difference in sample inclusion criteria, since individuals with systemic diseases were excluded from present study.

Atkinson and his co-authors [29] suggested that complaints of dry mouth may not reflect reduced salivary function and may instead reflect dehydration or other systemic condition. Therefore, authors considered that examining complaints of dry mouth will not reflect true risk for oral disease in the population. Similar results were reported among Iranian menopausal women with and without oral dryness feeling [30].

The present study showed that the subjects who had complaints had significantly less stimulated salivary flow rate than those who reported no complaint. The resting salivary rate was reduced in subjects with complaints but the severity of reduction was minor relative to stimulated flow rate. These findings are in agreement with that of Loesche et al [26] who found stimulated flow rate to be lower in those complaining of oral dryness. In contrast, a positive answer to the feeling of dry mouth according to Beck et al [31] and Bardow et al [19] seemed to be valuable for identifying patients with low resting salivary flow rates. Some authors found that dry mouth was not always associated with reduction in whole salivary output and therefore, insufficient mucosal wetting and changes in salivary composition have been regarded as factors that influence the perception of dry mouth [5,6]. These findings are supported by Dawes and Odlum [32] who found the mean residual volume of saliva is only reduced by 29% in patients with sever hyposalivation although they reported the feeling of a very dry mouth.

## Conclusion

• Dry lip was the commonest sign of oral dryness.

• Patients with dry lip had low salivary flow rate.

• Subjective complaints of oral dryness was prevalent in one fifth of the sample.

• Measurement of stimulated rather than resting salivary flow rate could be thought of as one of the diagnostic method for assessing dry mouth.

## Competing interests

The author(s) declare that they have no competing interests.

## Pre-publication history

The pre-publication history for this paper can be accessed here:


